# The Migratory Properties and Numbers of T Regulatory Cell Subsets in Circulation Are Differentially Influenced by Season and Are Associated With Vitamin D Status

**DOI:** 10.3389/fimmu.2020.00685

**Published:** 2020-05-19

**Authors:** Abigail A. Lamikanra, Hoi Pat Tsang, Shaza Elsiddig, Michael Spencer, Elinor Curnow, Robert Danby, David J. Roberts

**Affiliations:** ^1^National Health Service Blood and Transplant, Oxford, United Kingdom; ^2^Nuffield Division of Clinical Laboratory Sciences, Radcliffe Department of Medicine, University of Oxford, Oxford, United Kingdom; ^3^NHS Blood and Transplant, Statistics and Clinical Studies, Bristol, United Kingdom; ^4^Department of Haematology, Churchill Hospital, Oxford, United Kingdom; ^5^Anthony Nolan Research Institute, London, United Kingdom

**Keywords:** Tregs, regulatory T cells, vitamin D3, seasons, tolerance, migration

## Abstract

The control of peripheral immune responses by FOXP3^+^ T regulatory (Treg) cells is essential for immune tolerance. However, at any given time, Treg frequencies in whole blood can vary more than fivefold between individuals. An understanding of factors that influence Treg numbers and migration within and between individuals would be a powerful tool for cellular therapies that utilize the immunomodulatory properties of Tregs to control pathology associated with inflammation. We sought to understand how season could influence Treg numbers and phenotype by monitoring the proportion of natural thymus-derived Tregs (nTregs) defined as (CD3^+^CD4^+^CD25^+^FOXP3^+^CD127^–/lo*w*^) cells as a proportion of CD4^+^ T cells and compared these to all FOXP3^+^ Tregs (allTregs, CD3^+^CD25^+^FOXP3^+^CD127^–/lo*w*^). We were able to determine changes within individuals during 1 year suggesting an influence of season on nTreg frequencies. We found that, between individuals at any given time, nTreg/CD4^+^ T cells ranged from 1.8% in February to 8.8% in the summer where median nTreg/CD4 in January and February was 2.4% (range 3.75–1.76) and in July and August was 4.5% (range 8.81–3.17) *p* = 0.025. Importantly we were able to monitor individual nTreg frequencies throughout the year in donors that started the year with high or low nTregs. Some nTreg variation could be attributed to vitamin D status where normal linear regression estimated that an absolute increase in nTreg/CD4^+^ by 0.11% could be expected with 10 nmol increase in serum 25 (OH) vitamin D3 (*p* = 0.005, 95% CI: 0.03–0.19). We assessed migration markers on Tregs for the skin and/or gut. Here cutaneous lymphocyte associated antigen (CLA^+^) expression on CD25^+^FOXP3^+^CD4^+^/CD4^+^ was compared with the same population expressing the gut associated integrin, β7. Gut tropic CD25^+^FOXP3^+^β7^+^Tregs/CD4^+^ had similar dynamics to nTreg/CD4^+^. Conversely, CD25^+^FOXP3^+^CLA^+^Tregs/CD4^+^ showed no association with vitamin D status. Important for cellular therapies requiring isolation of Tregs, the absolute number of β7^+^CD4^+^CD25^+^FOXP3^+^Tregs was positively associated with 25(OH)vitamin D3 (*R*^2^ = 0.0208, *r* = 0.184, *p* = 0.021) whereas the absolute numbers of CLA^+^CD4^+^CD25^+^FOXP3^+^Tregs in the periphery were not influenced by vitamin D status. These baseline observations provide new opportunities to utilize seasonal variables that influence Treg numbers and their migratory potential in patients or donors.

## Introduction

Immune responses show considerable variation due to inherited and environmental factors, including not only acquired immune responses after exposure to specific antigens but also differences in individual innate responses and regulatory functions (1).

There are major patterns of development of these immune responses and functions over the lifetime of the individual, but there are also large and significant inter-individual and temporal variations (1–4). Although in principle, genetic and/or environmental factors must modulate the immune phenotype, a detailed description and quantitative understanding of the major factors involved in determining the inter-individual and temporal variations is far from complete [reviewed in ref. (5)].

CD4^+^CD25^*hi*^ and FoxP3^*hi*^T regulatory cells (Tregs) profoundly modulate effector responses, reviewed in ref. (6). In hematopoietic stem cell transplantation (HSCT) the proportion of Tregs/CD4^+^ T cells (or Tregs/CD3^+^ T cells) has a major effect on outcomes of allogeneic HSCT in animal models (7–9) and in observational studies in HSCT cohorts (10–12). In these studies, recipients of grafts with a higher than median proportion of Tregs had reduced incidence of acute graft versus host disease (aGvHD) (11) and non-relapse mortality, commonly due to complications associated with transplantation (12). Indeed, meta-analysis of observational outcomes from clinical cohorts shows that higher than median Treg/CD3^+^ T cell ratios in donor grafts are associated with reduced aGvHD, reduced transplantation-related mortality and improved overall survival (13). These initial clinical studies did not evaluate the role of naïve or memory Tregs, nor the role of Tregs that express skin-homing receptors in the graft, for example, cutaneous lymphocyte associated antigen (CLA) or gut-homing receptors such as α4β7 integrin, on HSCT outcomes. Furthermore, the factors that determine high Treg/CD4^+^ or high Treg/CD3^+^ T cell ratios or the number and proportions of naïve, memory or tissue-specific sub-sets of Tregs in donors and in donor grafts have not been described.

There is a wide individual variation in Treg numbers and Treg/CD4^+^ T cell ratios. For example, clinical studies have shown that Treg/CD4^+^ T cell ratios vary from 0.8 to 8.6% in donor HSCT grafts (12) and similarly the Treg/CD4^+^ T cells in peripheral blood can vary from 0.2 to 7% (14–16). There are also seasonal cycles of T effector cells associated with relapse of multiple sclerosis in the summer months (17) and reviewed in ref. (18) and significant differences of innate invariant natural killer T (i-NKT) cells that are associated with favorable outcomes following HSCT (19).

Some differences in Tregs are due to a seasonal variation in Treg/CD4^+^ ratios and expression of FoxP3 protein in humans which peak in the summer months and early autumn (June–September in the Northern Hemisphere) (2, 3, 6, 20, 21). These seasonal cycles in Treg numbers appear to follow the well-described high-summer peaks of vitamin D [see ref. (22) for review] and increased expression of the vitamin D receptor (23). Although 1, 25 dihydroxy-vitamin D may perpetuate the survival and activity of induced Tregs *in vitro* (24, 25), the studies of seasonal variation of vitamin D and Tregs have not resolved the association of vitamin D and Tregs in individuals.

Additional factors that vary with season and may also contribute to immune cell composition include urban particulate matter (PM) (26, 27) and high ambient temperature as evidenced in murine studies (28) and human studies (29). Cortisol, which forms part of the hypothalamic-pituitary-adrenal axis, is also sensitive to changes in daylight hours as well as the circadian clock, showing peaks in the first awakening hour and declining until the end of day (30–32).

We established a cohort of volunteers who were phenotyped regularly for Tregs and other immune cell types through the year to study the seasonal variation of immune cells, the numbers and phenotypes of Tregs, Treg/CD4 + ratios and the relationship of these immune-cell parameters with vitamin D3.

## Materials and Methods

All reagents were purchased from Sigma-Aldrich (United Kingdom) and antibodies raised in mice purchased from Becton Dickinson (BD, United Kingdom) unless stated otherwise.

### Recruitment and Sample Collection

The research protocol was approved by the National Research Ethics Committee (REC reference 15/NS/0060) and the National Health Service Blood and Transplant (NHSBT) Blood Supply CARE committee. All participants gave written informed consent in accordance with the Declaration of Helsinki. A proportion of whole blood from the sampling pouch was collected into K_2_EDTA vacutainer tubes (BD, Oxford, United Kingdom) and used to measure whole blood cell parameters (Sysmex XE-2100, Sysmex UK) and lymphocyte subsets and also to provide plasma. Samples were also saved for serum using vacutainer tubes for coagulating whole blood (BD). Whole blood parameters and the frequency of Tregs, Treg subsets and i-NKT cells were measured on the same day as sample collection. Plasma was collected from the upper layer following centrifugation of whole blood for 5 min at 1000 × *g* at room temperature, and then stored at −40°C. Serum aliquots were collected from supernatants following coagulation for 30 min in the dark at room temperature, centrifuged for 15 min at 1000 × *g*, stored at −40°C and then −80°C for long term storage after 4 months. To avoid confounding variations due to gender all data presented is from male donors.

### Immune Cell Phenotyping

Whole blood was lysed using red cell lysis buffer (15 mM Ammonium Chloride, 10 mM Sodium bicarbonate, 1 mM EDTA) with gentle agitation at room temperature for 10 min. Cells were washed 3 times in PBS by centrifuging at 500 × *g* for 5 min at 4°C, resuspended in PBS containing 0.5% BSA. Tregs were stained as follows: the cell surface phenotype was determined using an antibody cocktail that comprised BUV395 conjugated CD45 (clone H130), PE conjugated anti-human CD25 (clone M-A251), FITC conjugated anti-human CD3 (clone UCHT1), PerCpCy5.5 conjugated anti-human CD4 (clone RPA-T4) and PE-Cy7 conjugated anti CD127 (clone HIL-7R-M21). In some cocktails anti-CD127 was replaced with PE-Cy7 conjugated to anti-human CD45RA (clone HI100) or PE-Cy7 conjugated to anti-human CLA (clone HECA452) or biotinylated anti-human integrin β7 (clone FIB504, Biolegend) followed by 1 wash and incubation with PE-Cy7 conjugated to streptavidin (eBioscience). Cells were stained for 30 min at 4°C followed by wash in ice cold 5% BSA in PBS. Finally all samples were fixed and permeabilized in the cold with the FOXP3 staining buffer set (Miltenyi Biotech) before staining for FOXP3 using APC conjugated anti-human FOXP3 (clone PCH101) from eBioscience. NK T Cells and i-NKT cells were stained with a cocktail comprising APC conjugated anti-human CD56, PE conjugated anti-human Vα24, FITC conjugated anti-human CD3 (clone UCHT1) PerCpCy5.5 conjugated anti-human CD4 (clone RPA-T4). Before use, all antibodies were titrated for optimal working dilutions. CST bead calibration was used to normalize output from the flow cytometer to rule out batch effects due to changes in the performance of the flow cytometer. Samples incubated with the different cocktails were acquired in parallel with compensation controls and fluorescence minus one (FMO) controls for each fluorochrome using the LSR-II flow cytometer (BD). The gating used for analysis of Tregs, Treg subsets, NK and iNK T cells is outlined in Supplementary Figure 1. Absolute cell numbers of populations were determined using Truecount beads (BD) according to the manufacturer’s guidelines.

### Analysis of T Cells and Their Sub-Sets

All T cells were defined from viable DAPI negative events in the lymphocyte gate using the FSC vs. SSC plot, expression of CD45 and of CD3 (see Supplementary Figure 1). CD4^+^ T cells were defined from the same gate and CD8^+^ T cells estimated by subtracting the number of CD4^+^ events in the CD3^+^ gate from the total number of CD3^+^ events in the lymphocyte gate. Thymic derived nTregs were defined as CD45^+^CD3^+^CD4^+^CD25^+^FOXP3^+^CD127^–/*low*^, referred to as CD4^+^CD25^+^FOXP3^+^CD127^–/low^ or nTreg. The mostly naïve population of Tregs were captured using CD45^+^CD3^+^CD4^+^ CD25^+^FOXP3^+^CD45RA^+^ expression and referred to as CD45RA^+^Treg. The proportion of skin trophic and gut trophic Tregs were defined using the same T cell markers used for naïve Tregs and replacing the antibody specific to CD45RA with antibodies to CLA and β7, respectively. Restrictions in antibody availability, fluorochrome usage and cell numbers meant staining for the β7 integrin was the best option to estimate α4β7 gut associated Tregs since the frequency of all β7^+^Tregs was proportional to the α4β7 Tregs (Supplementary Figure 2).

### Measurement of Vitamin D Metabolites and Cortisol

Serum samples were thawed to room temperature in the dark. 25-hydroxyvitamin D [25 (OH)D] was measured in duplicate using an enzyme immune assay (EIA) and following the manufacturer’s guidance instructions (Immunodiagnostics, Tyne and Wear, United Kingdom). Cortisol was also measured in sera collected from the same donors and measured by EIA in duplicate following the manufacturer’s (R&D Systems) instructions. For each donor and analyte, measurements from different seasons were carried out on the same plate (i.e., samples from January and February versus those from June and July) with reference samples to control for plate variation.

### Acquisition of Environmental Data

Data for PM2.5 (particulate matter < 2.5 μm in diameter) and PM10 (particulate matter < 10 μm in diameter) was collected from Air Quality England^1^ and UK Air at the Department of the Environment, Fisheries and Rural Affairs, London: https://ukair.defra.gov.uk/data, accessed November 20, 2019. PM2.5 data for 2016 was not available in urban background or roadside sites in Oxford although PM10 data was available. Therefore PM2.5 and PM10 data reported here originates from urban stations in Oxford and the next closest station to the Oxford donor center in Reading that records this data. Data for temperature (mean monthly data) and total sunshine duration in Oxford (Location: 450900E 207200N, Lat 51.761 Lon −1.262, 63 m above mean sea level) were obtained from the meteorological office^2^.

### Statistical Analysis

GraphPad Prism version 7 (GraphPad Software, Inc., United States) and SAS/STAT version 9.4 (SAS Institute Inc., Cary, North Carolina, United States) was used to compare the mean variables in immune cells through one-way ANOVA with Tukey’s correction for multiple comparisons. Spearman’s two-tailed test (abbreviated as *r*) was used to assess associations between dependent and independent variables. The Mann–Whitney *U* test was used to compare ranks within each group. Unless otherwise stated median values with inter-quartile range (IQR) are reported in the text. Comparisons with a *p*-value of < 0.05 are reported as significant.

To explore whether winter and summer levels of nTreg were correlated, normal linear regression was used to describe the association between nTreg in January/February (winter) and July/August (summer). Results were adjusted for age at donation (Supplementary Figure 3). Mean nTreg was used for any donor with more than one nTreg measurement between January or February and December.

Normal linear regression was also used to describe the association between nTregs (in any month) and vitamin D3 level. Again, results were adjusted for age at donation. A random effect was included in this model to allow for correlation between repeat samples from the same donor. The mean of two duplicate measures of vitamin D for each donor sample was used in the analysis. All analyses were restricted to male platelet donors that were not taking vitamin D supplementation at the time of the study.

## Results

### Characteristics of Donors

**FIGURE 1:**
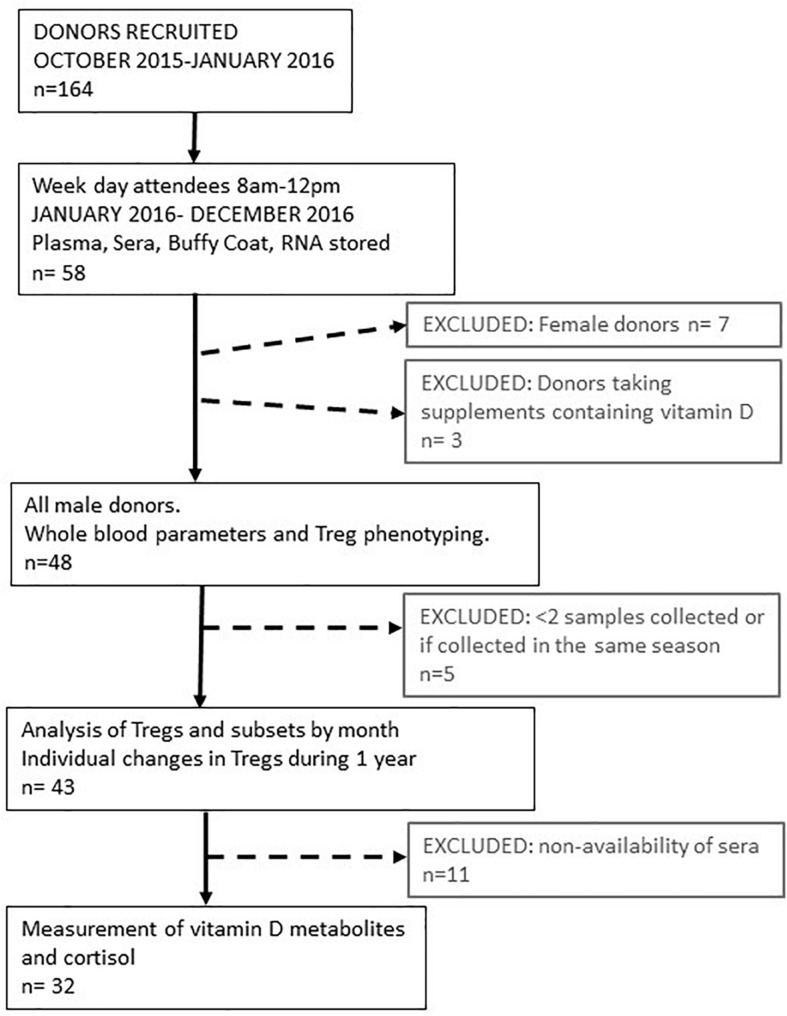
Schematic representing platelet donor recruitment and analysis of data. The median age range of all participants was 58 (range 21–72). The median number of samples taken during 1 year from each participant was 6 (range 2–9). Data obtained from these visits were pooled to provide an average value for parameters determined each month or every 2 months for 1 year. Treg and vitamin D3 metabolite variation of individual participants were determined in 32 of these donors where the median age was 59 years (range 23–72).

We initially recruited and consented 164 platelet donors that were healthy, eligible to donate and attended the platelet apheresis clinic between October 2015 and January 2016. Of these donors, a proportion regularly attended clinic in the morning. Samples were collected from 58 of those consecutive donors who attended clinic between January 2016 and December 2016, Monday to Friday inclusive with appointments before midday (1200). Collecting samples before midday each day facilitated the processing of samples on the same day and reduced the impact of diurnal changes on the variation of circulating immune cell numbers or characteristics (33). Figure 1 describes the process of recruitment and selection.

We initially followed 58 donors, 7 of whom were female. As so few samples could be collected from women, due to the bias of donors eligible for donating platelets, we only analyzed samples from male donors. We were able to resample 51 Caucasian male donors, 91% of whom were resampled at least three times during the year. Three of these donors were taking supplements that contained vitamin D and were subsequently removed from the dataset. So, we had samples from 48 donors to analyse for changes in immune cell numbers and phenotypes between each month of the year. Forty-three of these donors attended the clinic in different seasons so that their individual variation in immune cell frequencies could be assessed between seasons. Mean number of visits, 5 (95% confidence interval 5–6). Samples from 32 of these donors were available to determine serum levels of vitamin D metabolites and cortisol. Figure 1 summarizes how the samples from each participant were used.

### Monthly Variation of Immune Cells Over One Year

**FIGURE 2:**
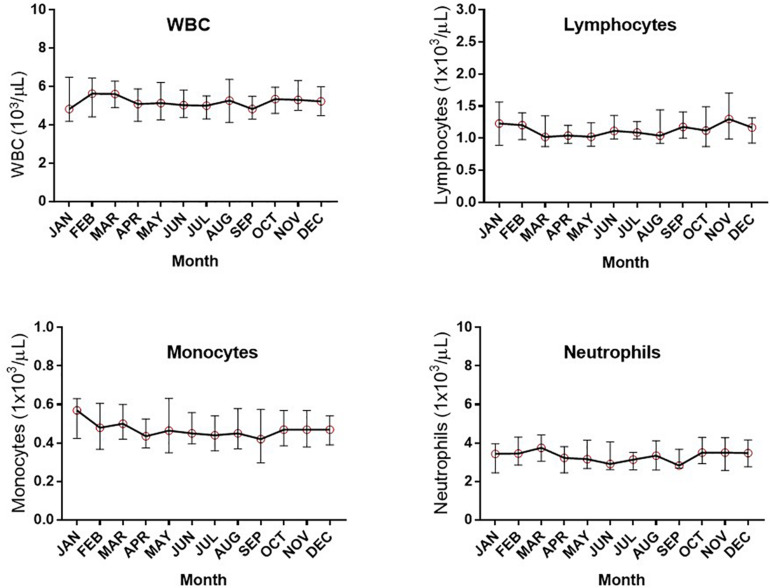
Monthly changes in whole blood parameters determined using an automated hematological analyzer. The variation in numbers of white blood cells (WBC), lymphocytes, monocytes and neutrophils are shown.

**FIGURE 3:**
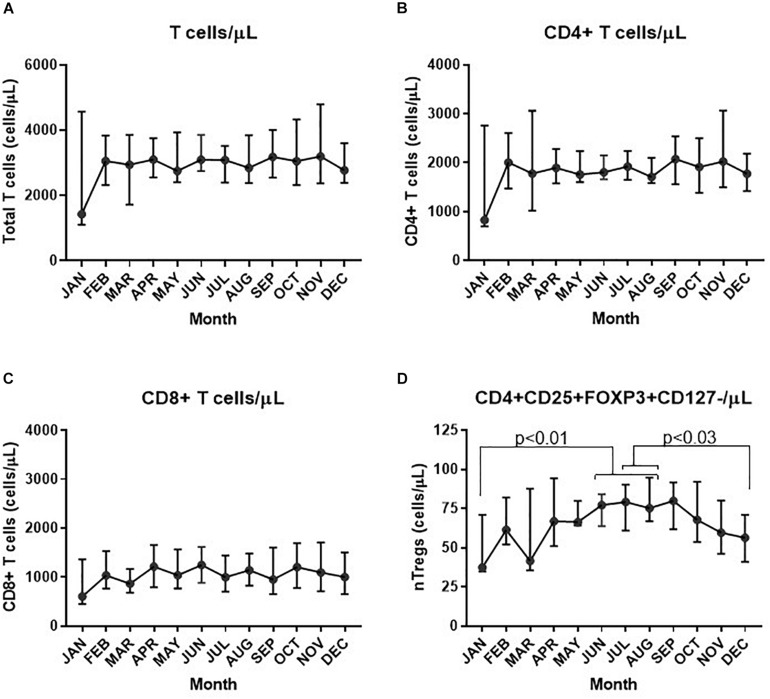
Variation in absolute numbers of T cells each month. The estimate of absolute numbers of T cell subsets are shown for each month and were determined after immune-phenotyping and analysis as described in Materials and Methods for all CD3^+^ T cells **(A)**, CD4^+^ T cells **(B),** CD8^+^ T cells **(C),** and nTregs **(D)**. In D the *p*-values are shown using one way ANOVA analysis of log transformed values where the *p*-values for changes in nTregs/μL between January and June, July and August are 0.010, 0.004, and 0.004, respectively; between July and December *p* = 0.032 and between August and December, *p* = 0.026.

Assessment of the absolute numbers of blood cells using the Sysmex analyzer did not reveal any seasonal change in the white blood cell count of neutrophils, monocytes or absolute numbers of lymphocytes (Figure 2). Similarly, the monthly changes in absolute numbers of all T cells, CD4^+^ and CD8 T cell determined by flow cytometry were not significant (Figures 3A–C). However, when the numbers of thymic-derived natural Tregs (CD4^+^CD25^+^FOXP3^+^CD127^–/low^) (nTregs) were assessed there was a gradual increase from January through to June, July and August that doubled the number of nTregs in circulation from a median of 37.4 nTregs/μl (IQR 71.0–34.9) to 77.3 nTregs/μl (IQR, 84.1–63.8), 79.2nTregs/μl (IQR, 90.3–61.0) and 75.2nTregs/μl (IQR, 91.7–61.8), *p* < 0.01. The decline in nTreg numbers from July and August to December (median of 56.5 nTregs/μl, IQR 70.8–41.0) was also apparent (*p* < 0.03) (Figure 3D).

We next assessed the FOXP3^+^ Tregs within both the CD4^+^ and CD8^+^ compartment (CD3^+^CD25^+^FOXP3^+^CD127^–/low^) and described as all Tregs from now on. We compared all Tregs/CD3^+^ T cells with nTreg/CD4^+^ T cells and with CD4^+^Vα24^+^/CD4^+^ T cells to represent a subset of immune-regulatory i-NKT cells.

**FIGURE 4:**
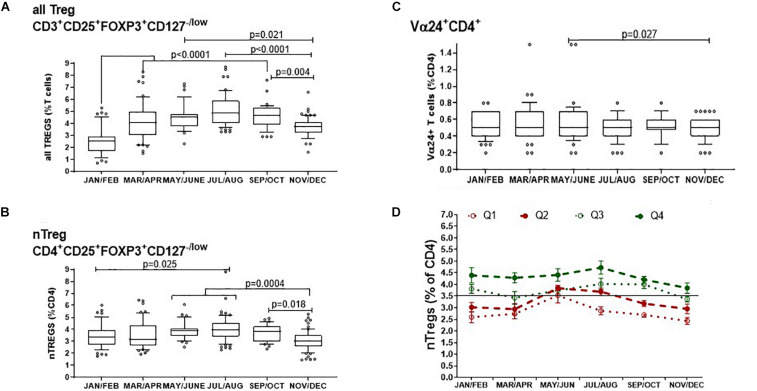
Bimonthly changes in immune-regulatory T cells between January and December of 1 year. The variation of **(A)** all FOXP3^+^ T cells that includes thymic and peripherally induced CD4^+^ and CD8^+^Tregs is compared with **(B)** non-activated CD25^*hi*^ nTregs and **(C)** with CD4^+^ T cells that express the Vα24 chain found on i-NKT cells. The *p*-values for non-transformed values following use of the ordinary one way ANOVA is shown where n is between 34 and 74 at each time point. Box and whisker bar graphs are used to show the median values with outliers lower than the 10th percentile and greater than the 90th percentile. **(D)** The proportions of nTregs in circulation vary between individuals as well as between seasons. The pattern of Treg levels in 1 year is shown for 43 donors that attended clinic multiple times in 1 year. Q1 represents donors that had mean nTregs less than the 25th percentile of annual nTregs and are compared with donors that had levels between the 25th and 50th percentile (Q2), between the 50th and 75th percentile (Q3) or above the 75th percentile (Q4) of nTregs in 1 year. Error bars show the standard error of the means at each time point.

The median proportion of all Treg cells/CD3^+^ T cells increased substantially from 2.5% (IQR, 2.9–1.7) in January and February to then almost double to 4.9% (IQR 6.0–4.0) in July and August (*p* < 0.001) after which they declined from September and October when they were 4.7% of CD3^+^ T cells (IQR 5.3–3.9), to 3.7% (IQR 4.1–3.2) in November and December (*p* = 0.004) (Figure 4A). Similarly, the proportion of nTreg/CD4^+^ T cells also increased from a median of approximately 3.4% (IQR 3.9–2.7) at the start of the year to a median of 4.0% (IQR 4.6–3.4) in July and August (*p* = 0.025) to then decrease back to 3.0% (IQR 3.5–2.6) at the end of the year (*p* = 0.004)(Figure 4B).

The proportion of CD4^+^T cells expressing the Vα24^+^ chain showed negligible seasonal change where median values were the same at 0.5% of all CD4^+^ T cells. A comparison of means showed that in May and June, the mean proportion of CD4^+^ T cells with the Vα24^+^ T cell chain was 0.58% and median was 0.5% (IQR 0.7–0.4). This then decreased in November and December where the mean of Vα24^+^ T cells was 0.47% but median was again 0.5% (IQR 0.6–0.4) *p* = 0.027 (Figure 4C).

Thus, the concentration of all FOXP3^+^ Tregs showed the greatest variation throughout the year of the typed sub-sets of peripheral blood mononuclear cells. We wanted to know if this was also true for the relative levels of nTregs in each participant as most literature refers to nTregs as *bone fide* Tregs. Figure 4D shows the distribution of nTregs in individuals that had annual nTreg levels below the 25th percentile (Q1), between the 25th and 50th percentile (Q2), between the 50th and 75th percentile (Q3) and above the 75th percentile (Q4). Those with average nTregs that fell below the annual median of 3.5% nTreg/CD4^+^ had nTreg frequencies that remained mostly below or at the 50th percentile throughout the year. The exception was between May and August where for those in which measurements were taken, nTreg/CD4^+^ increased by 1%. Conversely 50% of individuals with average nTregs above the 50th or 75th percentile started the year with nTreg/CD4^+^ between 4 and 4.5% that remained at or above median from April onward.

Moreover, using a linear regression model, we found that higher nTreg/CD4^+^ in winter was associated with higher nTregs in summer (*p* = 0.028). An increase in nTreg/CD4^+^ of 1% in January/February was associated with a 0.4% increase in nTreg/CD4^+^ in July/August (estimate, 95% confidence interval: 0.37%, 0.04–0.69%).

### Variation of Treg Subsets Over the Year

**FIGURE 5:**
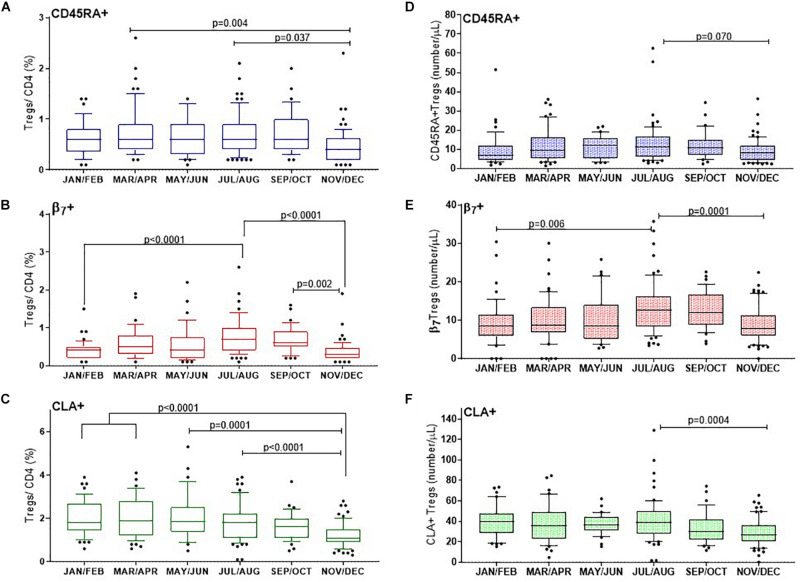
CD25^+^FOXP3^+^ Treg subsets show distinct changes through the year with differences in proportions of naïve and tropic marker expression. **(A)** CD45RA^+^ Tregs, **(B)** β7^+^ Tregs, **(C)** and CLA^+^ Tregs are shown as a proportion of CD4 + helper T cells. Absolute numbers of **(D)** CD45RA^+^ Tregs, **(E)** β7^+^ Tregs and **(F)** CLA^+^ Tregs are also shown. The ordinary one way ANOVA was used to determine *p*-values of non-transformed data and bar graphs with error bars to show outliers below and above the 10th and 90th percentiles, respectively.

We first looked at Treg subsets as a proportion of all CD4^+^ T cells as this analysis would allow assessment of their relative contribution to the total T helper cell population (Figure 5).

#### CD45RA^+^ Tregs

The CD45RA^+^ mostly naïve population of Tregs (CD3^+^CD4^+^CD25^+^FOXP3^+^CD45RA^+^) varied considerably between individuals and within certain months of the year. Nonetheless we found that on average the median proportion of these Tregs were at their lowest in November and December (0.4% CD45RA^+^Tregs/CD4^+^), IQR 0.6–0.2 compared with March and April (0.6%, IQR 0.9–0.4), *p* = 0.004 or July and August (0.6%, IQR 0.9–0.4), *p* = 0.037. See Figure 5A.

#### Tregs With Tissue Specific Homing Markers

In order to capture Tregs that could migrate to epithelial mucosa in the intestines, the contribution of (CD3^+^CD4^+^CD25^+^FOXP3^+^β7^+^) β7^+^Tregs to the CD4^+^ T cell population was determined. The proportion of β7^+^Treg/CD4^+^ T cells almost doubled from winter months (0.4%, IQR 0.5–0.2) to July, August (0.7%, IQR 1–0.4) *p* < 0.0001, with noticeably higher levels persisting in September and October (0.6%, IQR 0.9–0.5), *p* = 0.002 (Figure 5B). The median proportion of Tregs within the CD4^+^ compartment that expressed the skin trophic marker CLA (CD3^+^CD4^+^CD25^+^FOXP3^+^CLA^+^)/CD4^+^ T cells was unchanged from January and February (1.8%, IQR 2.7–1.4) until May and June (1.85%, IQR 2.5–1.4). CLA^+^Treg/CD4^+^ then declined from July and August (1.8%, IQR 2.2–1.1) through to the end of the year in November and December (1.1%, IQR 1.5–0.90), *p* < 0.0001 (Figure 5C).

We also assessed the absolute cell numbers of these subsets and observed that the median number of CD45RA^+^ Treg cells (CD3^+^CD4^+^CD25^+^FOXP3^+^CD45RA^+^)/μL were lowest in January/February (7/ μL, IQR 12–4) and in November/December (8/ μL, IQR 12–4) with a trend to peak in July and August (11 cells/ μL IQR, 17–6), *p* = 0.07 (Figure 5D). Similarly the median number of β7^+^ Treg cells (CD3^+^CD4^+^CD25^+^FOXP3^+^ β7^+^)/μL increased from January and February (9 cells/ μL IQR, 12–6) to July and August (13 cells/ μL IQR, 16–8), *p* = 0.006 to then decreased in November and December to 8 cells/ μL, IQR, 11–6), *p* = 0.001 (Figure 5E). In contrast the median number of CLA^+^ Treg cells (CD3^+^CD4^+^CD25^+^FOXP3^+^CLA^+^)/μL) were three to four fold greater in number than CD45RA^+^ or β7^+^ Tregs/μL and did not vary from January and February (at 39 cells/ μL IQR, 48–29) to July and August (39 cells/ μL IQR, 50–27). However, there was a significantly reduced number of the CLA^+^ subset by November and December (27 CLA^+^ Treg cells/μL, IQR 36–20, *p* = 0.0004).

#### Treg Subsets as a Proportion of Total Tregs

**FIGURE 6:**
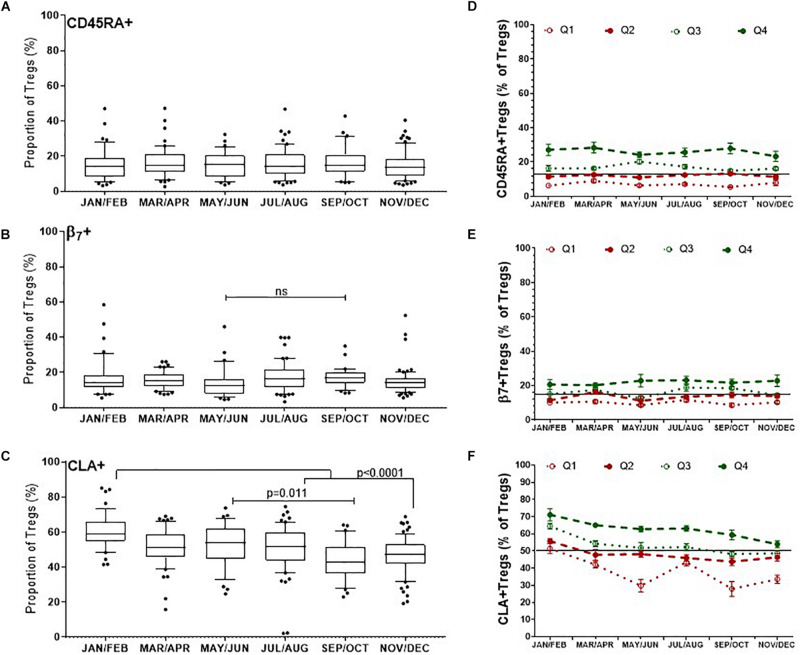
Differences in the dynamics of Treg subsets within the CD3 + CD4 + CD25^+^FOXP3^+^ (Total Treg) population. CLA^+^ Tregs are more likely to change substantially compared with CD45RA + and β7^+^ Tregs, where CLA^+^ Tregs have declined and β7^+^ Tregs may peak during late summer/autumn. Left side: Bimonthly variation of Treg subsets/Total Tregs determined between individuals **(A)** CD45RA^+^ Tregs, **(B)** β7^+^ Tregs, **(C)** CLA^+^ Treg. ns, *p* = 0.601. Right side: Bi-monthly changes in Treg subsets/Total Treg within individuals that are in each quartile **(D)** CD45RA^+^Tregs, **(E)** β7^+^Tregs, and **(F)** CLA^+^Tregs is shown. The mean ± SEM of Treg subset/Total Tregs is shown for donors that had an annual mean frequency of subset^+^ Tregs/Total Tregs below the 25th percentile (Q1) between the 25th and 50th percentile (Q2), between the 50th and 75th percentile (Q3) and above the 75th percentile (Q4).

There was no obvious change in the proportion of naïve CD45RA^+^ Tregs/(CD3^+^CD4^+^CD25^+^FOXP3^+^)Tregs, median values of which were between 14.5% and 15.4% (*p* = 0.9) (Figure 6A). Although there was a trend to increase β7^+^Tregs/Tregs between May/June (12.6%, IQR 16.4–7.9) and September and October (17%, IQR 20.25–13.9), this was not significant, *p* = 0.60 (Figure 6B). Conversely the median proportion of skin trophic CLA^+^ Tregs/Tregs decreased by almost 15% between January/February (59.1%, IQR 66.1–54.9) through to September/October (42.8%, IQR 51.6–36.5), *p* < 0.0001 (Figure 6C).

We assessed the same Treg subsets in each of the donors with multiple measurements in the same year. The mean annual value of Treg subset for each donor was used to assign each person to quartiles as described for nTregs in Figure 4D. Overall, individuals with CD45RA^+^ Tregs/Tregs and β7^+^Tregs/Tregs in the lowest two quartiles (Q1 and Q2) were more likely to remain relatively low throughout the year (Figures 6D,E) as were those with CLA^+^Tregs/Tregs (Figure 6F).

### Association of Seasonal Variables With Tregs

#### Vitamin D

**FIGURE 7:**
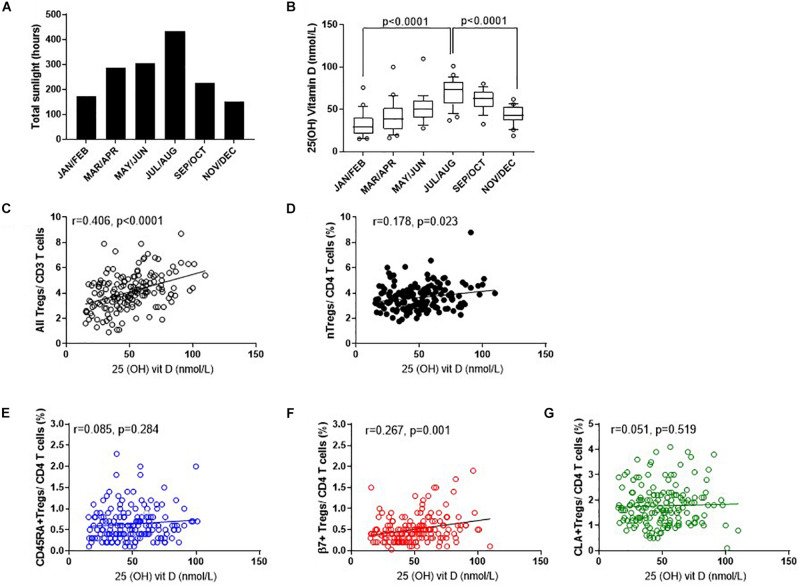
Seasonal variation of vitamin D status and Treg dynamics within the CD4 + helper population. **(A)** Daylight hours in Oxfordshire, **(B)** vitamin D status of donors attending clinic in the months shown. The association of 25 (OH) vitamin D metabolites with **(C)** all FOXP3^+^ T cells/CD3^+^ T cells *R*^2^ = 0.165, *r* = 0.406, *p* < 0.0001 **(D)** CD127^–/low^ nTregs/CD4^+^ *R*^2^ = 0.042, *r* = 0.178, *p* = 0.023. Also shown is the association of 25 (OH) vitamin D metabolites with **(E)** CD45RA^+^Tregs/CD4^+^ T cells *R*^2^ = 0.008, *r* = 0.085, *p* = 0.284, **(F)** with β7^+^Tregs/CD4^+^ T cells *R*^2^ = 0.067, *r* = 0.267, *p* = 0.001 and **(G)** with CLA^+^ Tregs/CD4^+^ T cells *R*^2^ = 0.0011, *r* = 0.051, *p* = 0.519. Box and whisker plots with error bars to show outliers below the 10th percentile and outliers above the 90th percentile.

To determine which factors may influence the observed differences in nTreg and Treg subsets we examined the role of endogenous vitamin D (Figure 7). Solar UVB irradiation mediates the conversion of 7-dehydrocholesterol to VD3 in the skin which is then converted to 25(OH)VD3 in the liver or in peripheral myeloid cells. Given that the number of daylight hours in the same year followed a similar distribution to the proportion of FOXP3^+^ Tregs (Figures 7A, 3, 4) we measured 25(OH) vitamin D metabolite in consecutive samples from donors that attended clinic and observed gradual increases from January through to September (Figure 7B). 25(OH) vitamin D was positively associated with all Tregs (*R*^2^ = 0.165, *r* = 0.406, *p* < 0.0001) and with thymic-derived nTregs (*R*^2^ = 0.042, *r* = 0.178, *p* = 0.023) (Figures 7C,D).

Conversely, there was no positive association between 25(OH) vitamin D and CD45RA^+^Treg/CD4^+^ (*R*^2^ = 0.008, *r* = 0.085, *p* = 0.284) nor CLA^+^Treg/CD4^+^ (*R*^2^ = 0.0011, *r* = 0.051, *p* = 0.519). See Figures 7E,G. However, β7^+^ Treg/CD4^+^ were positively associated with vitamin D status (*R*^2^ = 0.067, *r* = 0.267, *p* = 0.001) again relating seasonal changes of this subset of Tregs with observed changes in vitamin D status of this population (Figure 7F).

Data was adjusted for age and repeated measurements in the same donor as described in methods, and an association between nTreg/CD4^+^ and vitamin D (*p* = 0.0052) observed. An increase in vitamin D of 10 nmol was associated with an absolute 0.11% increase in nTreg/CD4^+^ T cell percentage (estimate, 95% confidence interval: 0.11, 0.03–0.19).

**FIGURE 8:**
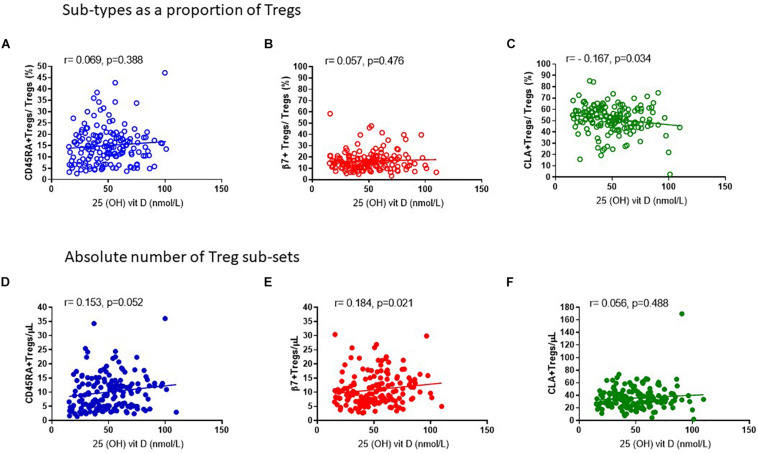
Vitamin D status is positively associated with the absolute number of CD45RA + and β7 + Tregs. The association of 25 (OH) vitamin D metabolites with **(A)** CD45RA^+^Tregs/Tregs, *R*^2^ = 0.008 *r* = 0.069, *p* = 0.388, **(B)** β7^+^Tregs/Total Tregs, *R*^2^ = 0.002, *r* = 0.057, *p* = 0.476 and **(C)** CLA^+^Tregs/Total Tregs, *R*^2^ = 0.029, *r* = –0.167, *p* = 0.034 is compared with the association of 25 (OH) vitamin D metabolites with absolute numbers/μL of **(D)** CD45RA^+^Tregs/μL, *R*^2^ = 0.021, *r* = 0.153, *p* = 0.052 **(E)** β7^+^Tregs/μL, *R*^2^ = 0.021, *r* = 0.184, *p* = 0.021, and **(F)** CLA^+^Tregs/μL, *R*^2^ = 0.010, *r* = 0.056, *p* = 0.488.

In keeping with the observation that the proportion of Tregs that are either CD45RA^+^ or β7^+^ appear mostly constant throughout the year, we observed no association of CD45RA^+^Tregs/Tregs (*R*^2^ = 0.008, *r* = 0.069, *p* = 0.388) nor of β7^+^ Treg/Tregs (*R*^2^ = 0.002, *r* = 0.057, *p* = 0.476) (Figures 8A,B) with 25 (OH) vitamin D3. In contrast, the CLA^+^ fraction of Tregs CLA^+^Tregs/Tregs was negatively associated with 25(OH) vitamin D in sera (*R*^2^ = 0.029, *r* = −0.167, *p* = 0.034) (Figure 8C). Conversely the absolute numbers of CLA^+^ Tregs (CLA^+^Treg cells/μL) did not change according to vitamin D status, indicating that the number of Tregs expressing CLA were not influenced by vitamin D (*R*^2^ = 0.010, *r* = 0.056, *p* = 0.488) (Figure 8F). However, the absolute numbers of β7^+^ Tregs were positively associated with vitamin D status (*R*^2^ = 0.021, *r* = 0.184, *p* = 0.021) as were CD45RA^+^Tregs (*R*^2^ = 0.021, *r* = 0.152, *p* = 0.052) (Figures 8D,E).

#### Cortisol

**FIGURE 9:**
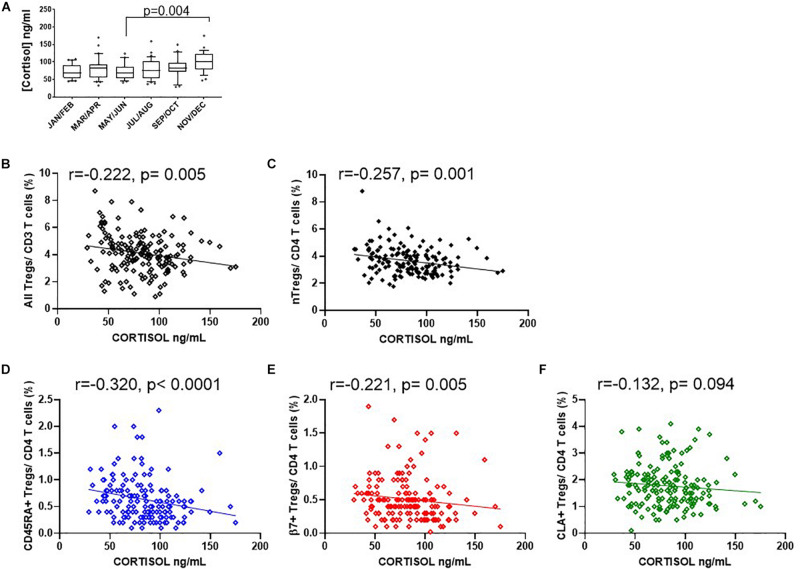
Seasonal variation of cortisol and Treg dynamics within the CD4+ helper population. **(A)** Bimonthly cortisol levels in sera collected between 08:30 am and 11:30 am. Also shown is the association of cortisol with **(B)** all FOXP3 + T cells/CD3 *R*^2^ = 0.041, *r* = –0.222, *p* = 0.005, **(C)** CD127^–/low^ nTregs/CD4 + *R*^2^ = 0.061, *r* = –0.257, *p* = 0.001, **(D)** CD45RA^+^Tregs/CD4^+^ T cells (*R*^2^ = 0.055, *r* = –0.320, *p* < 0.0001), **(E)** with β7^+^Tregs/CD4^+^ T cells *R*^2^ = 0.018, *r* = –0.221, *p* = 0.005 and **(F)** with CLA^+^ Tregs/CD4^+^ T cells *R*^2^ = 0.009, *r* = –0.132, *p* = 0.094. Box and whisker plots are with error bars to show outliers below the 10th percentile and outliers above the 90th percentile.

**FIGURE 10:**
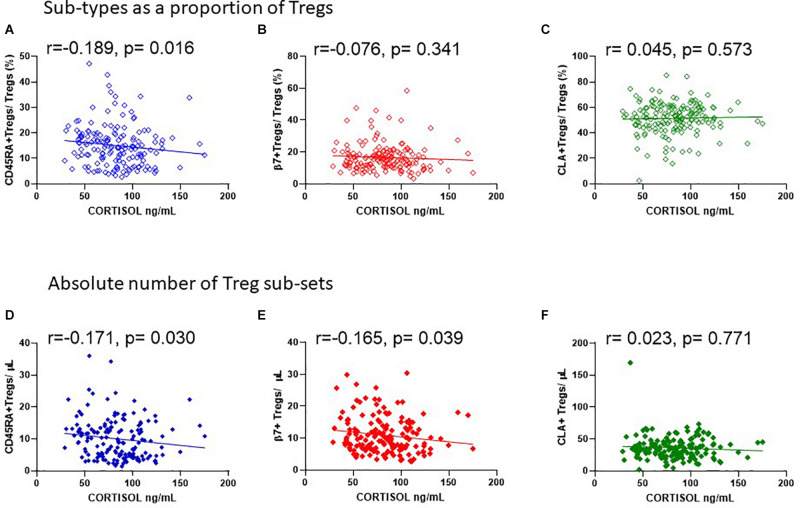
Cortisol levels in sera are negatively associated with the absolute number of CD45RA + and β7 + Tregs. The association of cortisol with **(A)** CD45RA^+^Tregs/Tregs, *R*^2^ = 0.016, *r* = −0.189, *p* = 0.016, **(B)** β7^+^Tregs/Tregs, *R*^2^ = 0.004, *r* = −0.076, *p* = 0.341, and **(C)** CLA^+^Tregs/Tregs, *R*^2^ = 0.001, *r* = 0.045, *p* = 0.573 is compared with the association of cortisol with absolute numbers/μL of **(D)** CD45RA^+^Tregs/μL, *R*^2^ = 0.020, *r* = −0.171, *p* = 0.030, **(E)** β7^+^Tregs/μL, *R*^2^ = 0.023, *r* = −0.165, *p* = 0.039, and **(F)** CLA^+^Tregs/μL, *R*^2^ = 0.005, *r* = −0.023, *p* = 0.771.

Since endogenous cortisol levels are influenced by sunlight hours, we also examined the association of total cortisol in each participant with the frequency of Tregs and their subsets. Cortisol was measured in samples from the same cohort that was used to measure 25(OH) vitamin D. We observed that the lowest levels of cortisol were in the summer months (May through to August) and highest levels in November and December (Figure 9A). Subsequently Cortisol in this cohort showed weak but significantly negative correlations with all FOXP3^+^ T cells/CD3 (*R*^2^ = 0.041, *r* = −0.222, *p* = 0.005) and with thymic-derived nTregs/CD4^+^ (*R*^2^ = 0.061, *r* = −0.257, *p* = 0.001) (Figures 9B,C). Similarly CD45RA^+^Treg/CD4^+^ and β7^+^ Treg/CD4^+^ were negatively associated with cortisol in sera (*R*^2^ = 0.055, *r* = −0.320, *p* < 0.0001 and *R*^2^ = 0.018, *r* = −0.221, *p* = 0.005, respectively) but CLA^+^Treg/CD4^+^ (*R*^2^ = 0.009, *r* = −0.132, *p* = 0.094) were not (Figures 9D–F). With the exception of CD45RA^+^Treg/Treg (*R*^2^ = 0.016, *r* = −0.189, *p* = 0.016) we observed no correlation between subsets as a proportion of Tregs (Figures 10A–C). However, the absolute numbers of CD45RA^+^ Treg and β7^+^ Treg cells/μL (*R*^2^ = 0.020, *r* = −0.171, *p* = 0.030 and *R*^2^ = 0.023, *r* = −0.165, *p* = 0.039, respectively) were weakly but significantly associated with cortisol (Figures 10D,E) whereas there was no correlation of CLA^+^Treg cells/μL (*R*^2^ = 0.005, *r* = −0.023, *p* = 0.771) with cortisol (Figure 10F).

## Discussion

Previous reports have described inter-individual variation of immune cells providing a rich resource to understand the influence of environment and genetic traits on different branches of immunity (3, 4). On the whole, the variation in T cell immunity and the cytokines that support their variability is largely governed by non-inheritable traits.

Here, we examined both inter-individual and intra-individual variation of FOXP3^+^ immuno-regulatory T cells and their subsets with changes in season in male platelet donors that returned to clinic on average five times a year. We found that both absolute numbers and the proportion of nTreg/CD4^+^ T cells were higher in the summer than in the winter. This was also true for allTreg/CD3 and was consistent with changes in vitamin D status and the ability of vitamin D to induce FOXP3 expression (24). In contrast similar changes to CD4^+^T cells expressing the Vα24^+^ chain (i-NKT/CD4^+^) were almost undetectable. Our data showing variation of nTreg within each donor from one season to the next allowed us to estimate that an increase of 10 nM 25 (OH) vitamin D3 in serum could result in a 0.1% absolute increase of nTreg as a percentage of CD4^+^ T cells. In this study we were able to monitor the intra-individual variation of Tregs to reduce any influence of genetic factors on our observations.

We also described changes in the CD45RA^+^ subset of FOXP3^+^ Tregs that can define the stability of Treg function. CD45RA has been used to isolate Tregs that retain immunomodulatory activity after expansion *in vitro* implying longer lived suppression *in vivo* (34, 35). We found that CD45RA^+^ Tregs/CD4^+^ remained at their highest levels between Spring and Autumn and represented less than 1% of the T-helper population at their peak. However, the proportion of CD45RA^+^ Tregs within the FOXP3^+^Treg population did not vary, so supporting previous observations that the apparent decrease in CD45RA^+^ Tregs/CD4^+^ T from Autumn to Winter is due to the increased proliferative potential of CD45RA-memory Tregs rather than a change in output from the thymus (36).

Examination of the potential of Tregs to migrate to gut mucosa (β7^+^Tregs) or to the skin (CLA^+^ Tregs) suggests that the dynamics of these two populations are affected differently by seasonal variables. The proportion of β7^+^Tregs within the T helper population resembled the month-to-month dynamics of nTreg/CD4^+^ and was positively associated with vitamin D status. Conversely, the dynamics CLA^+^Tregs/CD4^+^ differed considerably; between January and June CLA^+^Tregs/CD4^+^ and were on average twice the levels observed in November and December.

When we examined the composition of Tregs we found that the CLA^+^ Tregs/Tregs appeared to vary, whilst the proportion of β7^+^ and CD45RA^+^ within Tregs were mostly unchanged. This could in part be explained by the observed increase in absolute numbers of Tregs expressing the β7 integrin between January and July/August thereby reducing the proportion of CLA + cells in the Treg population which were unchanged during the same period (Figures 5E,F). The absolute numbers of Tregs expressing CLA did not change in association with vitamin D3 in sera whilst the number of Tregs expressing β7 integrin or CD45RA did change.

A recent study has shown that β7^+^ Tregs are CD45RA^+^ and comprise new emigrants from the thymus where expression of β7 is induced by IL-2 (37). CD45RA^+^ Tregs are considered most suitable for adoptive cellular therapy given that they retain suppressive function upon expansion (34, 38, 39). It is possible that the increase in β7^+^ Treg subsets is in part due to conversion of naïve β7^+^ CD4^+^ T cells to β7^+^FOXP3^+^ Tregs following increased exposure to sunlight and induced expression of VDR or similarly induced from the activated memory population of β7^+^CD4^+^ T cells (40).

We were unable to confirm CD45RA^+^ expression on CD3^+^CD4^+^CD25^+^FOXP3^+^CD127^–/low^β7^+^ Tregs (CD45RA^+^β7^+^ Tregs) nor assess the effect of season on CD45RA^+^β7^+^ Tregs in our cohort. However, analysis of this population in freshly isolated PBMCs from 6 donors shows that CD45RA^+^β7^+^ Tregs are on average 1/5th of β7^+^ Tregs (Supplementary Figure 4) indicating that the majority of β7^+^Tregs are likely effector memory Tregs and would be less stable if expanded *ex vivo*.

Brodin et al. (1) have also reported that the frequency of naïve T cells and their response to IL-2 is one of the few strongly heritable factors that can influence an immune response. The apparently positive but weak association of CD45RA^+^ numbers with vitamin D3 may also be dependent on the responsiveness of Tregs to IL-2 within each participant of this study. Nonetheless future application of season and vitamin D3 to enhance β7^+^Tregs will be dependent on establishing the stability of suppressive function of any of the induced Treg populations.

The variation in expression of CLA within the FOXP3^+^CD4^+^CD25^+^Treg population was considerable, both between and within individuals. The gradual intra-individual decrease from January through to mid-summer and November of the proportion of CLA + Tregs may be due to migration of CLA^+^ Tregs from the periphery to the skin in response to increased ultraviolet (UV) exposure from the sun (36, 41). This could again partly explain the marked decrease in CLA^+^Tregs/total Tregs in blood samples collected between March and September when exposure to sunlight had increased.

The poor recovery of CLA^+^ Tregs in samples collected when daylight hours were reduced in autumn and winter may be due to a delay in migration back to the periphery and also the decline in all Tregs since 1, 25 (OH) vitamin D can induce or stabilize FOXP3 (24) (U. Leuschner, personal communication) and with fewer daylight hours the potential to do so would be reduced.

Interestingly, a study of 350 older male and female participants aged between 69 and 80, has shown that the fraction of CLA^+^Tregs in circulating Tregs is positively associated with the degree of skin pigmentation following exposure to UV radiation from the sun in Florida (42). In this location daylight hours were between 800 and 500 hours from July 2014 to July 2015. This was at least double the number of hours in Oxford from January 2016 to December 2016 which were between 400 and 200 daylight hours.^3^ A correction for sex was applied to this data but data from male and female volunteers was not analyzed separately. Furthermore, the authors did not report vitamin D metabolites nor did they report exclusion of data from volunteers that were taking vitamin D supplements. Thus, whilst the data from this study provides compelling evidence for the ability of UV exposure to influence the migration of Tregs the conclusions presented therein are unlikely to apply to all demographics.

Other studies have also reported changes in Treg frequencies and phenotype with season using flow cytometry (4, 20, 21). None of these studies observed an association between Treg frequencies and vitamin D status nor did they relate vitamin D3 to Tregs within the same person during the course of 1 year.

The possible explanations for the different observations presented here include differences in the cohorts studied. Our data is from volunteers with an older mean age of 55 (median 58, range 21 to 72). Several studies have shown that age can influence the number and activation of different T cells especially baseline levels of Tregs and their naïve status [see Brodin et al for review (5)] (3, 4, 43). Also, we restricted our analysis to data from male volunteers to remove any confounding effects due to imbalance of genders since differences in hormones and the menstrual cycle can influence the relative proportion of Tregs in circulation (44) reviewed by (5). Variation of diurnal effects on Tregs (23) were reduced by collecting all samples between 8 am and 12 noon and measuring Treg numbers within 2 h of collection. Importantly, we defined the Treg population in our samples by selecting CD25^+^ FOXP3^+^CD127^–/low^ cells which would influence the specificity of the associations that we observed compared to studies that used different markers to stain for Tregs. Finally we were able to minimize the influence of genetic traits and age by periodically sampling from the same donors so that we could determine the association between nTreg and 25 (OH) vitamin D in each donor during 1 year.

A number of studies that utilize Mass Cytometry to define different Treg populations suggest that there can be overlap between Tregs and different Thelper cell subsets (45, 46) and these would not have been identified with the antibodies used in this study. Our study did not differentiate between effector memory and central memory Tregs. The use of additional phenotype markers to characterize the memory compartment in more detail using CD45R0, CD62L and specific chemokine receptor markers would have provided a more detailed understanding of the change in dynamics within the Treg memory and effector memory population.

Our inability to detect changes in the proportions of iNK T cells may have been due to the very low frequency of these cells in circulation and the reduced sensitivity of the approach we used to detect them which would require dual staining for Vβ11 TCR chain (47). Due to ethical considerations we were unable to measure the Treg levels and phenotype in the skin of healthy volunteers in this study to better explain the decrease in CLA^+^Tregs in peripheral blood.

We examined the association of vitamin D status on Treg frequencies as this would be the most tractable method to enhance Tregs in HSCT donors. However, environmental factors, other than vitamin D status and exposure to UV, are likely to contribute to the seasonal variability observed. Due to ethical considerations we were unable to retrospectively determine the exposure of individual participants to sun. Questionnaires to address this should be employed in future studies. External factors that could influence Treg frequencies include ambient temperature which shows the same pattern as sunlight hours and vitamin D status and is therefore also positively associated with Treg frequencies (see Supplementary Figure 5). Conversely PM2.5 and PM10 may negatively affect Treg levels (Supplementary Figure 6). In order to associate these observations more accurately with the Treg frequency of each donor the ambient temperature around the time of sample collection and PM levels local to each participant should also be measured.

We were however able to relate cortisol levels to Treg frequencies within participants that attended more than twice in 1 year. Here we observed negative associations with the frequencies of all Tregs and nTregs as well as absolute numbers of CD45RA^+^ and β7^+^ Tregs. This could explain the marked reduction of Tregs in November and December when cortisol levels were at their highest in this cohort.

There is evidence to support the interaction between the hypothalamus-pituitary axis, vitamin D3 and T cell frequencies. A high density of VDR and vitamin D3 metabolizing enzymes are present within the hypothalamus (48). Salivary cortisol can be reduced by 40% in healthy male and female volunteers following vitamin D3 supplementation with 2000 IU/day (*n* = 9) versus placebo (*n* = 6) for 14 days (49) and by 15% following supplementation of with 4,000 IU/day (*n* = 22) versus placebo (*n* = 19) for 16 weeks in relapsing and remitting patients with multiple sclerosis (50). However, these responses are likely to be most relevant during the first hour of awakening since the dip in cortisol over the course of 1 day remained unchanged between treatment and placebo (50).

Pre-clinical experimental models indicate that cortisol decreases Tregs through binding to the mineral corticoid receptor which enhances pro-inflammatory mediators [see ref. (51) for review]. Furthermore, the proportion of Tregs and their expression of FOXP3 is decreased in healthy volunteers whilst T helper 17 (Th17) cells are enhanced immediately after completing endurance exercise; and cortisol in serum is increased alongside IL-6, IL-10, and TGF-β. IL-6 and TGF-β are required to generate pathogenic Th17 cells and IL-6 in the sera of endurance-athletes may contribute to the reduced expansion of Tregs *in vitro* (52). These observations suggest that cortisol inhibits Tregs indirectly through enhancement of inflammatory mediators and/or support of Th17 expansion. Since the participants in our study were not taking vitamin D supplements, it is possible that the higher levels of cortisol compounded the low vitamin D status in November and December to reduce Treg numbers through enhancement of pro-inflammatory mediators.

Experimental models of Treg phenotype and expansion *in vivo* have shown that metabolites of vitamin A and short chain fatty acids can alter the migratory properties and numbers of Tregs reviewed in refs. (53) and (54, 55). In humans, exposure to allergens, different infections and the gut microbiome influence Treg numbers (3, 5) and seasonal variations in melatonin may influence Treg survival by inhibiting Th17 T cell development (17). The investigation of these possibilities in future studies would improve our understanding of how the balance of T regulatory cells can be modified for application in the clinic.

Treg numbers in HSCT grafts may vary from 0.8% nTreg/CD4 to 8.6% nTreg/CD4 and nTreg/CD4 frequencies above 3% can double overall survival (11, 12). Our observation that a 0.11% increase in nTreg requires a 10 nmol/L increase in VitD3 level indicates that to increase nTreg:CD4 from 2 to 3% (an increase of 1%) would require an increase of ≥91 nmol/L (i.e., [1/0.11^∗^10]) 25(OH) vitamin D3 in HSCT donors with Treg < 2%. A placebo controlled trial (56) suggests that a one-off dose of 250,000 IU vitamin D3 could safely increase 25 (OH) vitamin D3 by this amount (from 41.5 nmol/L to 103.2 nmol/L (CI 87.1–122.2 nmol/L) for 5–10 days during the winter months). This relationship may be modified in female donors and it will be important to establish this to better understand how to use vitamin D3 to induce Treg subsets.

In this study we have found that enhanced vitamin D status in healthy males was associated with changes in the frequency of different FOXP3^+^ Treg populations in the periphery that could be modified by levels of cortisol. An increase in the frequency of nTregs and β7^+^Tregs in the CD4^+^ T helper population and of FOXP3^+^ Tregs in the T cell population was more apparent in the summer months than in the winter months. The opposite was true of the proportion of Tregs in the periphery that expressed CLA as these were not associated with the vitamin D status of the cohort studied here. Since an increase in the frequency of circulating Tregs in the recipient that are CLA^+^ or α4β7^+^ is associated with reduced risk of skin or gut GvHD, respectively (57, 58) the baseline data presented here will support endeavors to improve clinical outcomes following HSCT. Further investigations into how appropriate supplementation with vitamin D3 could modify cortisol levels, the migratory potential and suppressive stability of Tregs in HSCT are therefore warranted.

## Data Availability Statement

The datasets generated for this study are available on request to the corresponding author.

## Ethics Statement

The studies involving human participants were reviewed and approved by North of Scotland Research Ethics Service (NHS Grampian). The participants provided their written informed consent to take part in this study.

## Author Contributions

AL designed and implemented the study, analyzed data, prepared figures, and wrote the manuscript. HT performed the experiments and analyzed the data. SE and MS performed the experiments. EC performed the statistical analysis of data. RD contributed to the design of the study. DR conceived, designed the study, and wrote the manuscript.

## Conflict of Interest

The authors declare that the research was conducted in the absence of any commercial or financial relationships that could be construed as a potential conflict of interest.

## Footnotes

1 https://www.airqualityengland.co.uk/local-authority/?la_id=267

2 https://www.metoffice.gov.uk/pub/data/weather/uk/climate/stationdata/oxforddata.txt

3 http://www.timebie.com/sun and https://www.timeanddate.com/sun accessed December 2018.
