# Supplementary material for: The Migratory Properties and Numbers of T Regulatory Cell Subsets in Circulation Are Differentially Influenced by Season and Are Associated With Vitamin D Status
Source: Front Immunol. 2020 May 19;11:685. doi: 10.3389/fimmu.2020.00685 (PMC7248210; doi:10.3389/fimmu.2020.00685)

Supplementary Figure 1:

(a) Gating used to quantify relative proportions of FOXP3^{+} T cells and nTregs.

(i) to (ii) steps used to gate based on size and to remove doublets. (iii) CD45+ gate excludes non lymphocytes.

(iv)CD3+ gate to select all T cells. (v) FOXP3+ gate to select all (CD4+ and CD8+) FOXP3+ T cells that are CD127-/lo.

(vi)Selection of CD3+CD4+ gate. (vii) Selection of CD25+CD127$^{-/lo}$ CD4+ T cells. (viii) FOXP3+CD25+ cells are selected from the CD4+CD25+CD127$^{-/lo}$ population.

CD127+ signal in all lymphocytes (relative to cells in P7 and P12) is shown inset.

Solid arrows denote selection of populations within each gate. Broken arrows, back gating of FOXP3+T cells to confirm CD4 expression.

Bi-exponential logarithmic scales are used for plots to differentiate T cell populations and display events that occur at or below zero.

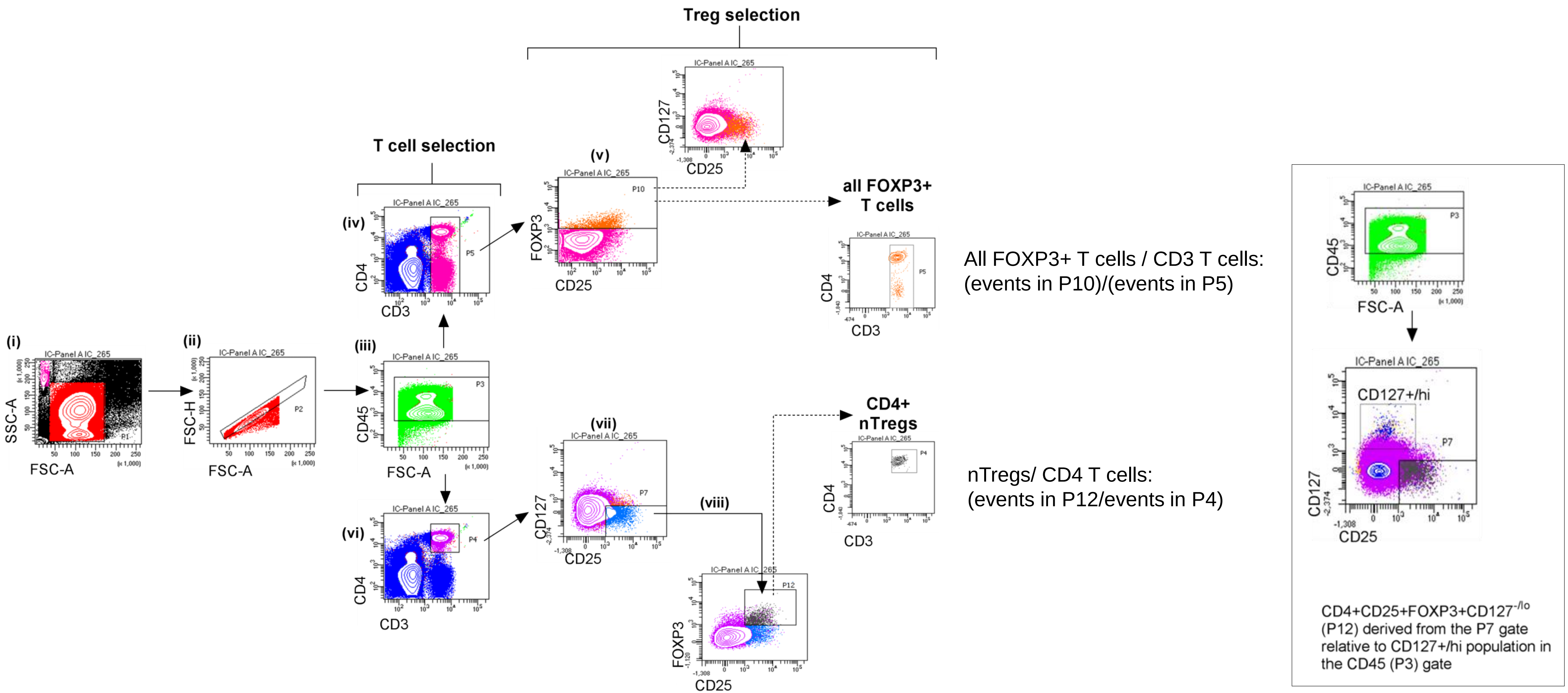

Supplementary Figure 1:

(b) Gating used to quantify relative proportions of sub-types within the Treg population follows on from CD45 selection in Supplemental Figure 1a
(i)Selection of CD4 T cells and then (ii) FOXP3+CD25hi to (iii) determine the proportion of Tregs expressing sub-type markers CD45RA, CLA and β7 integrin.
FMO controls are shown below each step (iv) FMO- PerCpCy5.5, (v) FMO- FITC, (vi) FMO-APC, (vii) FMO-PE, (viii) FMO-PE-Cy7.

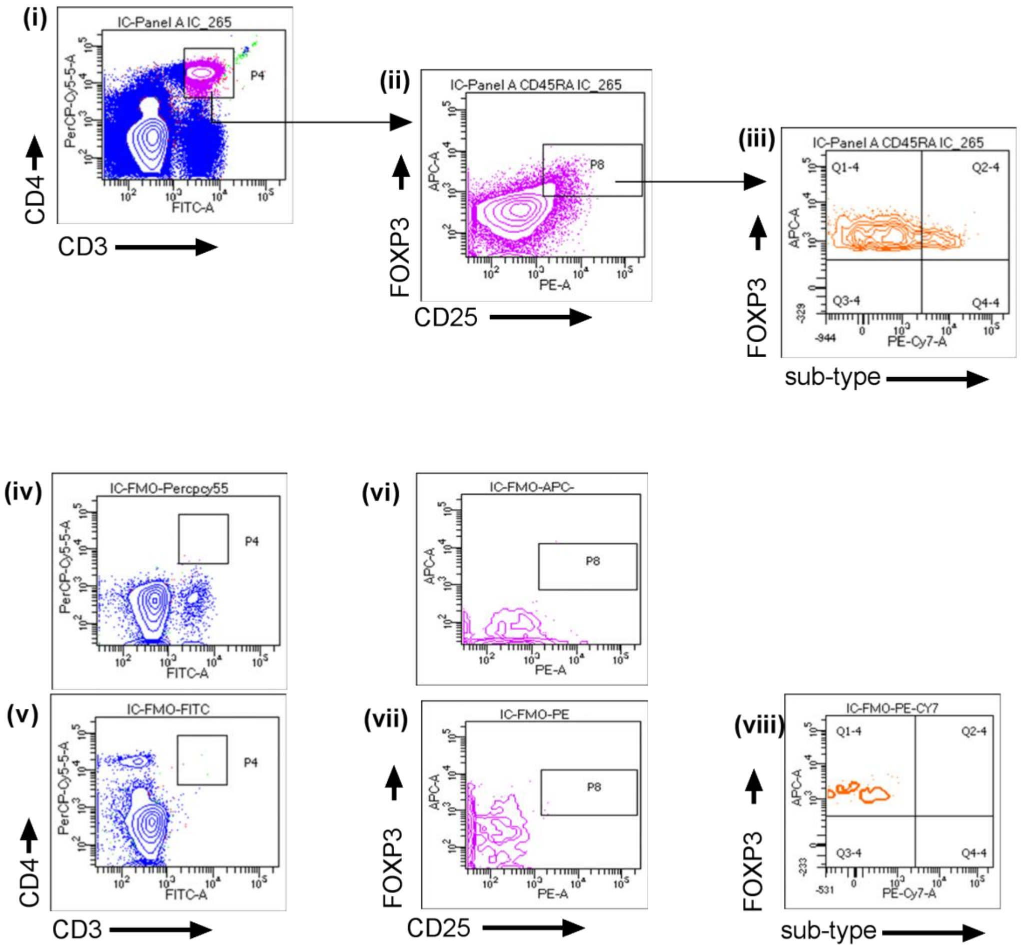

Supplementary Figure 1:

(c) Gating used to quantify T cells that express the variable alpha chain 24.

(i)Selection of lymphocyte gate and then (ii) exclusion of DAPI positive dead cells to select (iii) viable CD3+ T cells that are (iv) CD4+ Vα24 positive.

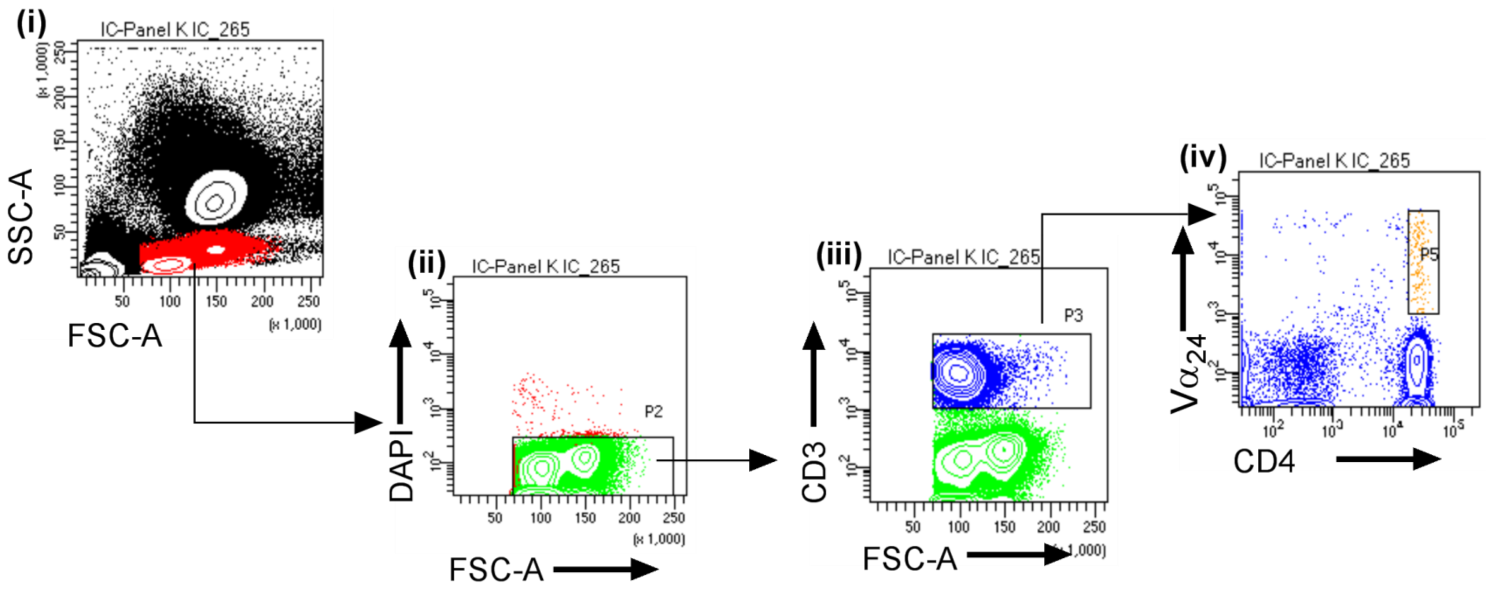

Supplementary Figure 2.
Staining for β7+ Tregs is proportional to the smaller fraction of gut associated Tregs that express α4β7 integrins

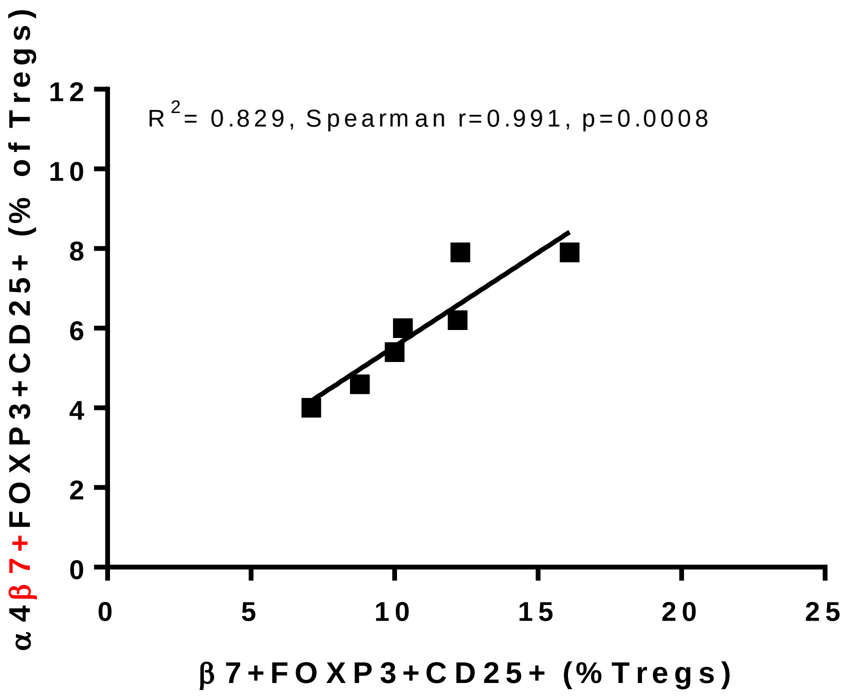

Supplementary Figure 3. The age and collection of samples from each donor is shown every 2 month (A) and the mean ± SD is shown in (B)

A)

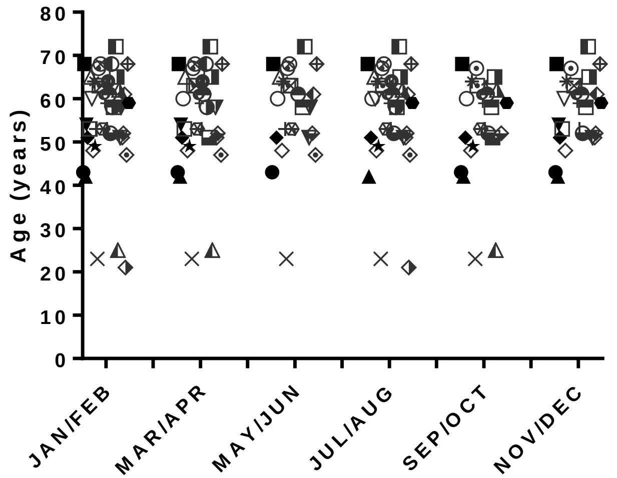

B)

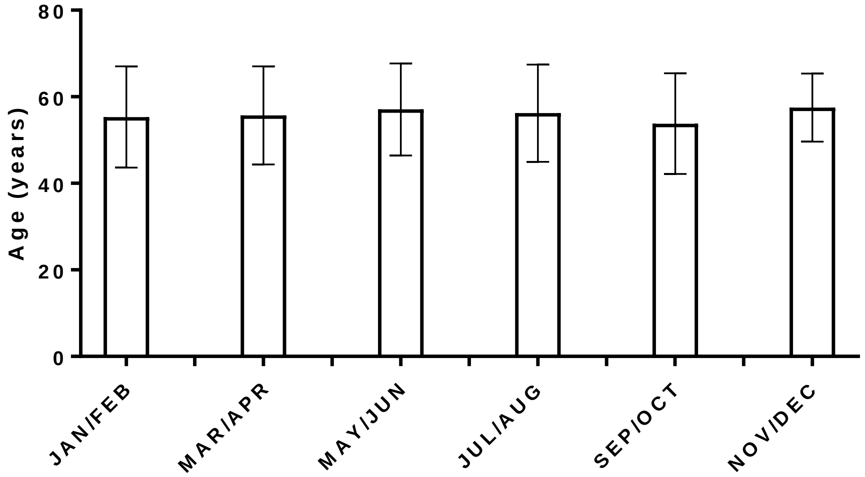

Supplementary Figure 4. The majority of β7+ Tregs in the periphery are CD45RA negative. (A) Representative analysis of CD45RA expression on β7+ nTregs in PBMCs isolated from lymphocyte cones prepared from 6 anonymous donors is shown. Lymphocyte cones were stained 1 to 2 days after collection during January and February. (B) Bar graph shows the average measurements from 6 donors of β7+CD45RA+ nTregs/CD4 (Q2-1) and β7+CD45RA- nTregs/CD4 (Q1-1). The p value following a paired one tailed Wilcoxon test is shown.

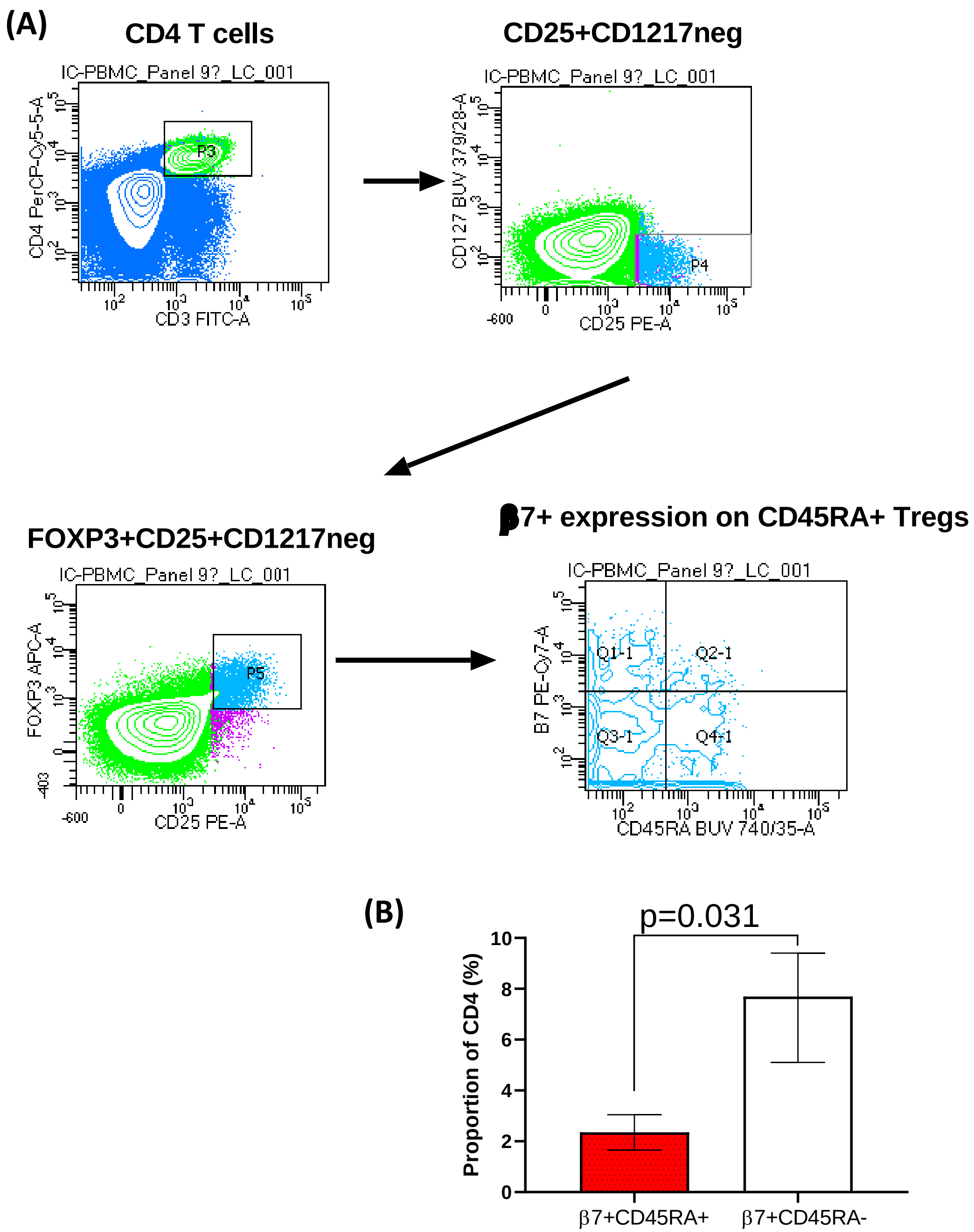

Supplementary Figure 5. (A) Ambient temperature in Oxford City Centre 2 miles from the study site. (B) The association of ambient temperature with Treg frequencies determined each month for male donors that attended the study site. See donor characteristics in the manuscript for more details. Average minimum temperatures and maximum temperatures for each month were obtained from https://www.metoffice.gov.uk/pub/data/weather/uk/climate/stationdata/oxforddata.txt.

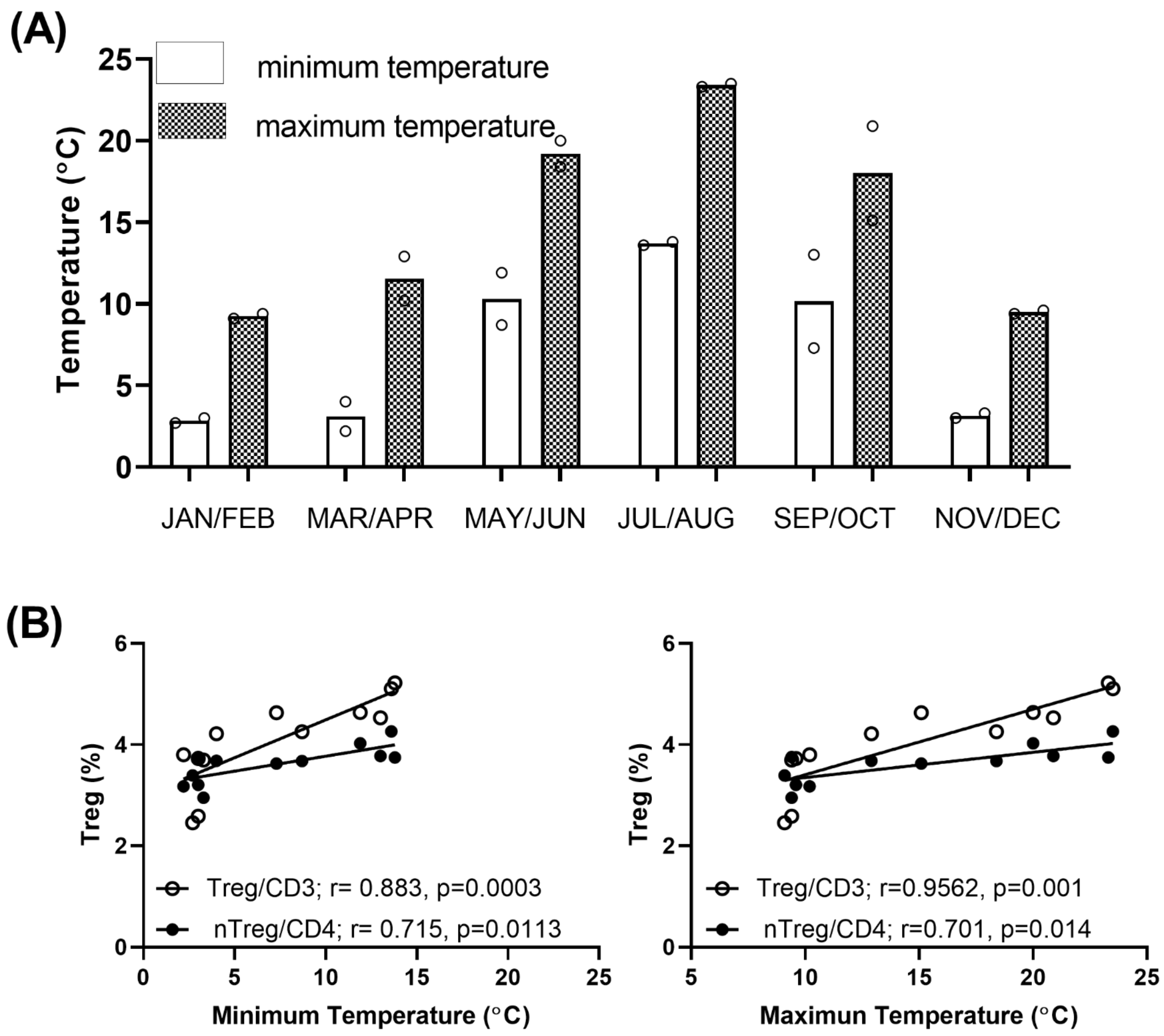

Supplementary Figure 6. Particulate matter (PM) measurements at sites local to the study site attended by platelet donors and association with Treg frequencies. (A) PM data during the study period recorded for Oxford City Centre (site 1, 2 miles from the study site) and in Reading City Centre (site 2, 25miles from the study site) are shown to estimate PM exposure for participants in the study. Correlations between monthly PM readings and the average monthly Treg frequencies are shown for (B)PM2.5 and (C)PM10.

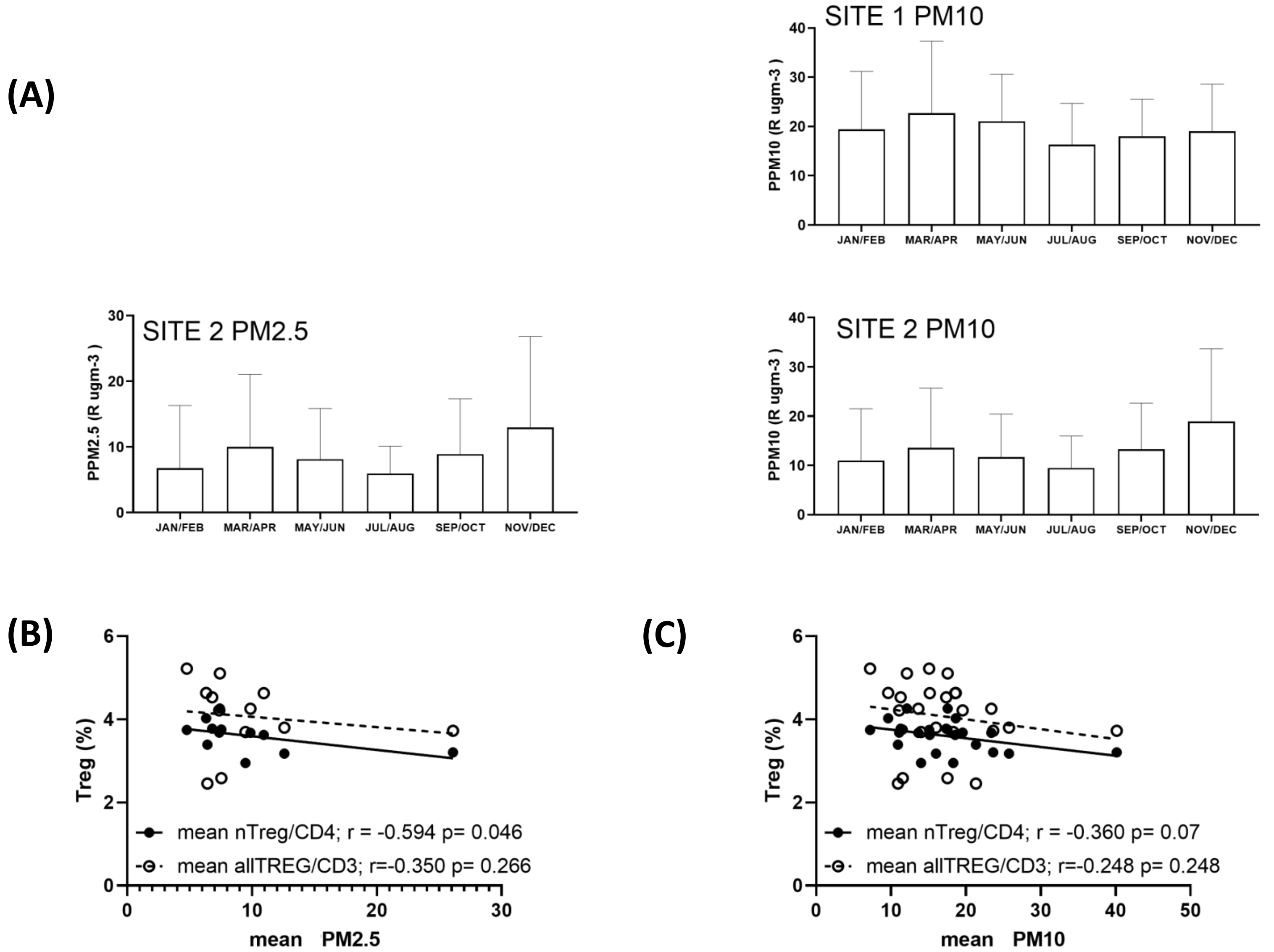

Supplement: Supplementary file 1 [file Data_Sheet_1.PDF]
